# Prevalence and associated factors of female genital mutilation among Somali refugees in eastern Ethiopia: a cross-sectional study

**DOI:** 10.1186/1471-2458-9-264

**Published:** 2009-07-27

**Authors:** Getnet Mitike, Wakgari Deressa

**Affiliations:** 1 School of Public Health, Addis Ababa University, P. O. Box 9086, Addis Ababa, Ethiopia

## Abstract

**Background:**

Eastern Ethiopia hosts a substantial number of refugees originated from Somalia. Female genital mutilation (FGM) is a common practice in the area, despite the campaigns to eliminate it.

**Methods:**

A cross-sectional study was conducted among 492 respondents sampled from three refugee camps in Somali Regional State, Eastern Ethiopia, to determine the prevalence and associated factors of FGM. Data were collected using pre-tested structured questionnaires.

**Results:**

Although the intention of the parents to circumcise their daughters was high (84%), 42.4% of 288 ≤12 girls were reported being undergone FGM. The prevalence increased with age, and about 52% and 95% were circumcised at the age of 7–8 and 11–12 years, respectively. Almost all operations were performed by traditional circumcisers (81%) and birth attendants (18%). Clitoral cutting (64%) and narrowing of the vaginal opening through stitching (36%) were the two common forms of FGM reported by the respondents. Participation of the parents in anti-FGM interventions is statistically associated with lower practice and intention of the procedures.

**Conclusion:**

FGM is widely practised among the Somali refugee community in Eastern Ethiopia, and there was a considerable support for the continuation of the practice particularly among women. The findings indicate a reported shift of FGM from its severe form to milder clitoral cutting. More men than women positively viewed anti-FGM interventions, and fewer men than women had the intention to let their daughters undergo FGM, indicating the need to involve men in anti-FGM activities.

## Background

Despite international and national efforts to eliminate the practice, female genital mutilation (FGM), also known as female genital cutting, is widely practiced throughout much of Africa mainly in relation to social, cultural and religious reasons [1,2]. It is an old-age practice believed to be existed in the central Africa, Egypt and the Middle East. Between 100 and 140 million girls and women worldwide are estimated to have undergone the practice of FGM [2]. According to the World Health Organization (WHO) estimates, more than three million girls mainly in Africa are estimated to be circumcised each year [2]. Estimated prevalence of FGM in 27 countries across Africa ranged from 98% in Somalia to less than 1% in Uganda [2].

Female genital mutilation comprises all procedures involving partial or total removal of the external female genitalia without medical reasons [2]. Accordingly, the WHO has classified it into four types [2] namely: Type I – partial or total removal of the clitoris, with or without excision of part or all of prepuce (clitoridectomy), Type II – partial or total removal of the clitoris and the labia minora, with or without excision of the labia majora (excision), Type III – narrowing the vaginal opening through the creation of a covering seal by cutting and repositioning the labia minora and/or the labia majora, and with or without removal of the clitoris (infibulation), and Type IV – all other harmful procedures to the female genitalia for non-medical reasons, such as pricking, piercing, incising, scraping and cauterizing the genital area. In some areas particularly in north-east Africa, Type I is most often referred to as "*sunna"*, while Type III (infibulation) is known as "*pharaonic"*, the former comprising the majority of milder forms of FGM. A less extensive form of infibulation, Type II, is referred to as "intermediate FGM".

A recent WHO collaborative study in six African countries (i.e., Burkina Faso, Ghana, Kenya, Nigeria, Senegal and Sudan) revealed 24% Type I, 27% Type II and 23% Type III among 28,393 women presented for delivery at 28 obstetric centres [3]. Somalia (98%), Djibouti (93%), Eritrea (89%) and Ethiopia (74%) are the main countries in east Africa where the practice of FGM is widespread [2].

Although the achievement so far has not been as desired, Ethiopia has made an important progress towards the reduction of FGM. According to the 2000 and 2005 Demographic and Health Survey (DHS) reports of Ethiopia, the prevalence rates for FGM were found to be 80 and 74%, respectively [4,5], indicating that the practice is deep-rooted and declining slowly. The practice is most common in the eastern parts of the country, where prevalence ranges from 85 to 97%. In the country's 2005 DHS report, about 70% of the women interviewed were in favour of the discontinuation of the practice [5], compared with 40% in the 2000 DHS [4].

Interest for eradicating the practice of FGM has increased in the last two decades [6]. More recently, a variety of community-based anti-FGM interventions have been implemented in many parts of Ethiopia. A reproductive health (RH) project based on community-based behaviour change approach was started in 1997 in three refugee camps in Somali Regional State of Ethiopia by Save the Children United Kingdom (SC-UK), Administration for Refugee and Returnee Affairs (ARRA) for Ethiopia, and the Somali Regional State Health Bureau (SRSHB). High prevalence of FGM, very low contraceptive use and unsafe motherhood were among the major gaps that justified the implementation of the project. The aim of the project was to contribute to the improved quality of integrated RH information and services for Somali refugees and refugee affected communities, and to finally contribute to the national effort to mobilize support for the eradication of FGM and other harmful traditional practices in the region.

The main activities of the project included sensitisation and mobilization of the community through Information, Education and Communication/Behaviour Change Communication (IEC/BCC) toward RH issues focusing on harmful traditional practices, particularly FGM, through training of the community members such as community health agents (CHAs), elders, religious and traditional leaders, women with key roles in the community, establishment of anti-FGM committee and community action team, and provision of training and improving quality of RH services through the camp-based health centre. One of the approaches of the project was to emphasize the shift in the practice of FGM from severe to its milder forms. However, baseline information and systematic documentation was not carried out when the program was initiated. The aim of this study was, therefore, to determine the reported prevalence of FGM in a refugee community, and to investigate the socio-economic factors that influence the practice among parents of young girls. The findings might be helpful for the final evaluation of the project.

## Methods

### Study area and population

A community-based cross-sectional study to determine the prevalence and associated factors of FGM was carried out in February 2004 in three Somali refugee camps in the Somali Regional State in Eastern Ethiopia. Among the 11 regions in the country, the Somali region (with ≈4.5 million people) ranked first (97%) in the number of women who have undergone FGM, infibulation or Type III (62%) being its most common form [5]. Jijiga is the administrative capital of the Somali Regional State. This part of Ethiopia hosted a huge number of refugees originated from Somalia, following the ravaging civil war that occurred in that country during the late 1980s and early 1990s. Although there were about 150,000 refugees from Somalia in the region in 2000, the repatriation program conducted between 2001 and 2003 significantly reduced their number. As a result, at the time of the survey there were over 27,000 refugees in the main three camps (52% in Aysha, 43% in Kebribeyah, and 5% in Hartishek). The refugee camps had their own health centre administered by the ARRA.

### Sample size and sampling methods

The respondents for this study were married women in the reproductive age group (15–49 years) and married men 15 years and above. A minimum sample size of 460 was calculated using Epi Info 2002 software package population survey formula using 50% prevalence with 5% margin of error at 95% confidence level after considering 20% non-response rate. The sample size was equally divided between women and men, and was distributed to the three refugee camps proportional to their population size. Households with eligible study participants were selected by systematic sampling method through house-to-house visits. Girls 1–12 years of age and living in the selected households were listed based on the reports of the parents, and interviewed about their practice with FGM. From each selected household, either the wife or husband was interviewed, to avoid the double counting of girls. Additionally, local female circumcisers were identified for interview through purposive sampling.

### Data collection

Data were collected from the respondents by six (three females and three males) trained local data collectors who completed 12^th ^grade education and had recent experience in data collection on reproductive health surveys. The interviewers were residents of the area, knew the culture and language of the people. A nurse supervisor was also employed from Kara Mara Hospital in Jijiga town to facilitate, coordinate and supervise the fieldwork. Two days intensive training for data collectors and a supervisor was conducted at Jijiga SC-UK office. The training focused on thorough familiarization with the questionnaire, interview techniques, household selection and filling of the questionnaire.

Data were collected using a structured individual household interviewer-administered questionnaire initially prepared in English and then translated into *Amharic*, the national language. The questionnaire used in this survey addressed socio-demographic characteristics, knowledge and perceptions about FGM and its types, complications related to FGM, sources of information about the practice, age of girls at which they undergo the practice, and any intention of circumcision of a daughter. The respondents were interviewed at home by interviewers of the same sex with local Somali language (i. e., *Somaligna*) and the responses filled into the final *Amharic *questionnaire.

The questionnaire was pre-tested in a non-refugee village around Jijiga town. In order to assess the recent practice of FGM in the refugee community, the parents were asked about the status of their last two daughters in the household who were between 1–12 years of age. Data collection was entirely based on self-report and no inspection of genitalia was performed to assess the consistency between self-report and observed circumcision. A separate structured questionnaire prepared for traditional female circumcisers was also administered by the nurse supervisor.

### Data analysis

Data generated from the study were entered into Epi Info version 6.0 and transferred to SPSS version 11 for analysis. Proportions, means, tables and graphs were used for data summarization and presentation. Degree of association was measured by odds ratio with 95% confidence interval (CI). Logistic regression was performed for analysing practice of FGM and intention to circumcise after controlling for age of the daughters, sex, educational status and other socio-economic factors.

### Ethical considerations

The technical proposal for this study was reviewed and approved by the committee from Save the Children UK in Addis Ababa. Official letters were written to the respective ARRA offices and the refugee camps to allow for the implementation of the survey. Informed consent was obtained from the camp leaders and study participants after being informed in detail about the nature and the purpose of the study. All respondents were interviewed separately and appropriate measures were taken to assure confidentiality of information both during and after data collection.

## Results

A total of 492 respondents and 26 traditional female circumcisers participated in the study, with a response rate of 97%. All respondents were Muslims and belonged to the Somali ethnic group originated from Somalia due to the early 1990s civil war. As shown in Table 1, 45.1% of the respondents were from Aysha refugee camp, followed by Kebribeyah (42.7%) and Hartishek (12.2%). Two hundred and forty-six (50%) of the respondents were females. About 91% of the females and 57% males did not attend any formal education. The majority of the respondents (75.2%) were engaged in polygamous marriage, and 85% were housewives. About 67% of the men reported that they did not have any specific job. The majority of male (85.8%) and female (86.2%) respondents lived in the refugee camps for more than 10 years.

**Table 1:** Socio-demographic characteristics of the study participants in refugee camps, Jijiga, 2004.

Variable	Male, n (%)	Female, n (%)	Total, n (%)
Camp			
Aysha	111 (45.1)	111 (45.1)	222 (45.1)
Kebribeyah	105 (42.7)	105 (42.7)	210 (42.7)
Hartishek	30 (12.2)	30 (12.2)	60 (12.2)
Age in years			
18 – 24	30 (12.2)	22 (8.9)	52 (10.6)
25 – 34	49 (19.9)	63 (25.6)	112 (22.8)
35 – 44	72 (29.3)	118 (48.0)	190 (38.6)
≥44	95 (38.6)	43 (17.5)	138 (28.0)
Formal schooling			
Yes	105 (42.7)	22 (8.9)	127 (25.8)
No	141 (57.3)	224 (91.1)	265 (74.2)
Educational status			
Read and write	15 (14.3)	6 (27.3)	21 (16.5)
Primary (1–8)	53 (50.5)	13 (50.1)	66 (52.0)
Secondary (9–12)	34 (8.4)	2 (9.1)	36 (28.3)
12^th ^grade and above	3 (2.8)	1 (4.5)	4 (3.2)
Marital status			
Married (monogamous)	201 (81.7)	169 (68.7)	370 (75.2)
Married (polygamous)	42 (17.1)	57 (23.2)	99 (20.1)
Divorced/widowed/separated	3 (1.2)	20 (8.1)	23 (4.7)
Occupation			
Housewife	0 (0.0)	209 (85.0)	209 (42.5)
Jobless	165 (67.1)	12 (4.9)	177 (36.1)
Student	7 (2.9)	0 (0.0)	7 (1.4)
Teacher	17 (6.9)	2 (0.8)	19 (3.9)
Retail trade	15 (6.1)	19 (7.7)	34 (6.9)
Religious leader	3 (1.2)	1 (0.4)	4 (0.8)
Circumciser	1 (0.4)	1 (0.4)	2 (0.2)
Other	38 (15.4)	2 (0.8)	40 (8.2)
Additional income			
Yes	39 (15.9)	31 (12.6)	70 (14.2)
No	207 (84.1)	215 (87.4)	422 (85.8)
Duration of stay in the camp			
≤ 10 years	35 (14.2)	34 (13.8)	71(14.4)
> 10	211 (85.8)	212 (86.2)	423 (85.6)

Total, N (%)	246 (50.0)	246 (50.0)	494 (100.0)

The age group and the reported prevalence of FGM among 288 girls between 1–12 years of age included in the study are presented in Table 2. Of the total girls, 178 (61.8%) were second from the last (older) and 110 (38.2%) were the youngest (last daughter) for their parents. About 30% were less than five year-old, 55.6% between 5–10 year-old and 14.6% between 11–12 years of age. Of 288 girls, 122 (42.4%) had had FGM. Of girls between 7–12 years, about 73% had undergone FGM. The prevalence increased with age, and at the age of 7–8 years, about 52% of the girls had already undergone the procedure, and by the age 11–12 years, about 98% had experienced FGM.

**Table 2:** Age specific prevalence (%) of self-reported FGM among all girls ≤ 12 years old in refugee camps, Jijiga, 2004.

Birth status of the girls	Age group (years)						
		
	1–2	3–4	5–6	7–8	9–10	11–12	Total
Second from the last (older)							
FGM, n (%)	0	0	8 (29.6)	21 (53.8)	29 (78.4)	39 (95.0)	97 (54.5)
Sub-total, n	12	23	27	39	37	40	178
Last girl (younger)							
FGM, n (%)	0	1 (3.4)	1 (4.8)	11 (47.8)	10 (76.9)	2 (100.0)	25 (22.7)
Sub-total, n	22	29	21	23	13	2	110

Both girls							
FGM, n (%)	0	1 (1.9)	9 (18.8)	32 (51.6)	39 (78.0)	41 (95.2)	122 (42.4)
Total, n	34	52	48	62	50	42	288

Ninety-seven (54.5%) of the second from the last (older) and 25 (22.7%) of the younger daughters had undergone FGM at the time of interview (Table 2). More specifically, among 116 older girls ≥7 years, 77% had undergone the procedure compared with 60.5% of 38 younger girls in the same age group. Girls ≥7 years were significantly more at risk of FGM than girls aged less than 7 years (P < 0.0001, OR = 32, 95% CI 15–72). The mean age of FGM for the second from the last girls (older) was 7.5 (± 1.7) years, and the corresponding age for the last (younger) girls was 6.5 (± 1.4) years, with the median age of FGM being 8 and 7 years, respectively. The optimum age at which the practice of FGM is being initiated in the study community was likely to be between 5–8 years old. Girls above 7 years of age were at higher risk of FGM than younger girls, indicating that the prevalence increases with age. A total of 166 girls had not undergone FGM at the time of the study, the major reason being under age for FGM (91.8%), followed by a plan to undergo the procedure (6%).

The distribution of different forms of FGM reported in this study and the operators is shown in Table 3. The most prevalent form of FGM, as reported by the respondents, was partial or full clitoral cutting (63.9%). This form of FGM was higher in the younger girls (80.0%) than the older daughters (59.8%). The majority (81.1%) of the operations of FGM had been performed by traditional local female circumcisers, followed by traditional birth attendants (TBAs).

**Table 3:** Distribution of the different forms of FGM and its operators in refugee camps, Jijiga, 2004.

	Younger girls, n (%)	Older girls, n (%)	Total, n (%)
Type of FGM			
Clitoral cutting	20 (80.0)	58 (59.8)	78 (63.9)
Vaginal sewing	5 (20.0)	39 (40.2)	44 (36.1)
Operator			
Traditional female circumciser	19 (76.0)	80 (82.5)	99 (81.1)
Traditional birth attendant	6 (24.0)	16 (16.5)	22 (18.0)
Health worker	0 (0.0)	1 (1.0)	1 (0.9)

Total	25 (20.5)	97 (79.5)	122 (100.0)

Knowledge of the respondents about various forms of FGM practiced in the refugee camps was assessed, and most (67.1%) reported clitoral cutting and vaginal sewing (25.2%). The respondents were also asked about their intention of subjecting their daughters to FGM in the future. About 84% of the respondents replied that they had an intention that their daughters should undergo FGM. Men and women had statistically significant difference in their intentions, the intention among women to circumcise the girls was higher (91.1%) than that reported by men (75.2%) (P < 0.001, OR = 3.4, 95% CI 1.9–6.2). Knowledge about the complications of FGM was also asked and it was consistently below 50% for both men and women in four major complication areas such as excessive bleeding (46.7%), obstructed labour (45.5%), menstrual disturbance (39%) and sexual problems (35.8%). Knowledge about the relationship between FGM and excessive bleeding was statistically higher among women (46.7%) than men (30.1%) (P < 0.001, OR = 2.04, 95% CI 1.4–3.0).

Respondents were also asked on the sources of information about the complications of FGM. Anti- FGM committee, refugee health centre and training on anti-FGM activities were mentioned as the major sources of information about possible immediate and long-term risks of health complications associated with FGM. In addition, many women respondents explained their personal experience as a source of knowledge about its complications. Study participants were also asked about their involvement in the campaigns and activities against FGM. Generally, a little less than a third of the interviewees had participated in the anti-FGM interventions. About 89% of men and 55% of women participants positively viewed the usefulness of the anti-FGM interventions.

A total of 26 female circumcisers participated in the study, of whom 10 (38.5%) were from Kebribeyah, 9 (34.6%) from Hartishek and 7 (26.9%) from Aysha refugee camps. We totally identified 46 circumcisers from all refugee camps. Although refusals were very low, four circumcisers in Aysha refugee camp refused to participate in the study as they did not support the anti-FGM campaign. The age of the circumcisers ranged from 30 to 70 years with a mean (± SD) age of 45.05 ± 10.1, and 50% of them being within the age group of 40 – 49 years. Most of the circumcisers were illiterate (73.1%), involved in monogamous (54.8%) and polygamous (26.9%) marriages, widowed/divorced (19.2%), often involved in FGM (84.6%) and TBA (80.8%) activities. The majority did not have other sources of income (65.4%) except the food ration supplied by UNHCR/ARRA. About 46% of them reported that they practiced FGM for more than five years.

The forms of FGM commonly practiced by the circumcisers included clitoral cutting (11.5%), vaginal stitching (42.3%), and both clitoral cutting and stitching (46.2%). About 77% (n = 20) of them were performing FGM during the survey mainly for generating their livelihood. Only six (23.1%) circumcisers had discontinued the practice of FGM at the time of the study ascribed to be due to the anti-FGM training they received. Of the interviewed circumcisers, 10 (37.4%) received training on anti-FGM activities for 5–7 times, followed by 3–4 times (30.8%), but 5 circumcisers reported that they did not participate in any type of anti-FGM training. Five circumcisers participated in any kind of anti-FGM activities both as leader and member of the campaigns, of whom three had discontinued the practice of FGM. The survey also assessed the practice of circumcisers in the use of any type of anaesthesia during circumcision, and 15 (57.7%) of them reported the use of local anaesthesia, and almost all of them purchased it from local drug vendors. However, 11 (42.3%) did not use any type of anaesthesia and the main reasons given were its unavailability or lack of knowledge about its use. A razor blade was the most commonly used instrument to operate the procedures of FGM.

On interview about the current attitude of the circumcisers about the forms of FGM they would like to perform, 25 (96.2%) preferred to perform the milder clitoral cutting. In addition, they were also asked about the possible complications due to FGM, and reported birth complications (84.6%), menstrual problems (84.6%), excessive bleeding (53.8%) and problems during sexual intercourse (46.2%). Some of them also mentioned infections and infertility as problems associated with FGM.

When logistic regression was applied using forward stepwise (Likelihood Ratio) analysis, the practice of FGM was significantly associated with age of the parent and their involvement in anti-FGM interventions (Table 4). The practice of FGM was more reported among younger parents <35 years (adjusted OR = 6.7, 95% CI 2.6–16.7), while less practice was reported among parents who participated at least in one of the anti-FGM activities (adjusted OR = 0.3, 95% CI 0.2–0.6). No statistically significant association was found between the practice of FGM and the duration of residence in the camp, educational status of the parent or knowledge of the major complications related to the practice. Intention to circumcise a daughter was significantly associated with sex of the respondents and their participation in anti-FGM interventions (Table 5). Being male (adjusted OR = 0.28, 95% CI 0.15–0.55) and being involved in anti-FGM interventions (adjusted OR = 0.56, 95% CI 0.42–0.98) were associated with low intention to practice FGM.

**Table 4:** Logistic regression for factors associated with the practice of FGM among girls in refugee camps, Jijiga, 2004.

Parent characteristics	Circumcision status of girls		Odds Ratio (95% CI)*	
	
	Circumcised, n(%)	Not circumcised, n(%)	Crude	Adjusted
Age of parent				
< 35	54 (87.1)	55 (52.9)	6.01 (2.5, 15.94)	6.65 (2.6, 16.7)**
≥35	8 (12.9)	49 (47.1)	1	1
Duration of stay in camp				
≤10 years	5 (8.1)	16 (15.4)	0.5 (0.1, 1.5)	1.06 (0.3,3.6)
>10	57 (91.9)	88 (84.6)	1	1
Attended formal school				
Yes	55 (88.7)	98 (94.2)	2.1 (0.6, 7.9)	3.6 (0.9, 14.4)
No	7 (11.3)	6 (5.8)	1	1
Knew at least one of FGM complications				
Yes	16 (25.8)	27 (26.0)	1.1 (0.5, 2.3)	0.5 (0.2, 1.2)
No	46 (74.2)	77 (74.0)	1	1
Participation in anti-FGM interventions				
Yes	28 (45.2)	77 (74.0)	0.3 (0.2, 0.6)	0.3 (0.2, 0.6)**
No	34 (54.8)	27 (26.0)	1	1

* CI = Confidence interval, ** = Statistically significant difference

**Table 5:** Distribution of factors affecting intention to circumcise and results of logistic regression, refugee camps, Jijiga, 2004.

	Intention of the parents		Odds Ratio (95% CI)*	
	
Parent characteristics and exposure	Circumcise, n (%)	Not circumcise, n (%)	Crude	Adjusted
Age of parent				
<35	197 (47.9)	42 (54.5)	0.77 (0.47, 1.25)	0.74 (0.42, 1.32)
≥35	214 (52.1)	35 (45.5)	1	1
Sex				
Male	185 (45.0)	57 (74.0)	0.29 (0.16, 0.51)	0.28 (0.15, 0.55)**
Female	226 (55.0)	20 (26.0)	1	1
Duration in the camp				
≤10 years	57 (13.9)	12 (15.6)	0.87 (0.43, 1.82)	0.83 (0.39, 1.73)
>10	354 (86.1)	65 (84.4)	1	1
Formal education				
Yes	90 (21.9)	35 (45.5)	0.34 (0.20, 0.50)	0.66 (0.35, 1.25)
No	321 (78.1)	42'(54.5)	1	1
Participation in anti-FGM interventions				
Yes	106 (26.8)	29 (40.3)	0.54 (0.31, 0.94)	0.56 (0.32, 0.98)**
No	290 (73.2)	43 (59.7)	1	1

CI = Confidence interval, *** = Statistically significant difference

## Discussion

In this study, the reported prevalence of FGM and its different forms was studied among young girls ≤12 years of age in order to get a good understanding of its current practice in the area. Our study was conducted in refugee communities originated from Somalia from late 1989 to 1996 and residing in Eastern Ethiopia. More than 98% of the women in Somalia have undergone FGM [1], most of whom were subjected to the severe form of FGM (infibulation) and girls usually undergo the practice between 5–12 years old [7].

About 42% of all the girls identified through the present survey were reported as being subjected to FGM, and almost all undergone the procedure before the age of 12 years. FGM is initiated at the age between 6–8 years, and beyond this age, the probability of subjecting a girl to FGM increased significantly, suggesting its persistence widespread practice among the study community. In a study conducted in Egypt, 80% of girls were circumcised between the ages of 5–9 years old [8] and 20% of girls between 4–9 years old in Sudan had undergone FGM and 50% of their guardians indicated that it would be performed later [9].

The practice of FGM is widespread in Eastern Ethiopia. In the Ethiopia DHS 2005, about 97% of the women in the age of 15–49 years old interviewed from Somali region had been exposed to FGM [5]. The present findings also highlight the universal practice of FGM in Somalia although the study population might not be representative of the population in the home country with respect to FGM. The practice of FGM in many societies is considered as an obligatory social and traditional norm mainly to maintain virginity, sexual chastity, and to reduce and control female sexuality [1,10,11].

This study also shows the forms of FGM performed on the girls, with 64% clitoral cutting and 36% vaginal stitching (infibulation). The lower percentage of the latter figure might show that this type of FGM is becoming less common, but it is difficult to compare our findings with other studies since it might not be representative of the general population in Somalia or Ethiopia. In the 2005 DHS report of Ethiopia, 84% of the circumcised women from Somali region undergone infibulation [5]. The lower proportion of infibulation in our sample might also be attributable to under-reporting of the procedure due to the current anti-FGM intervention carried out in the area. In contrast to our study, the findings from Sudan indicate that infibulation was performed in 67% of girls aged 4–9 years who had undergone FGM [9].

Many studies have cited the importance of tradition and culture in addition to religion as the major driving forces behind the practice of FGM [11,12]. However, there is no scriptural evidence in the religious support of FGM [1]. The religious dimension of FGM is complicated as it is interwoven with cultural and social aspects of the society [11]. People try to relate the milder forms of FGM (Type I and Type II) with Islamic religion, while infibulation (Type III) was said to exist even before the advent of Islam. And hence, resisting and eradicating infibulation could not be challenging compared to Type I and II (*Sunni*) FGM.

It is believed that FGM is one of the deeply rooted traditional practices, which has a mixed influence from culture as well as religion. All these aspects are highly linked together in Eastern Ethiopia and are very difficult to consider them separately. Many people do not easily understand the distinctions between the religion, culture and the practice of FGM. In a study conducted in Eastern Ethiopia, religion has been found to be one of the main motives for performing FGM among Muslim community [13]. This necessitates the strengthening of national laws against FGM that seeks the support of religious and community leaders in the campaign to fight the practice, and this would be helpful in convincing those who support it.

The long persistence of FGM is partly ascribed to the dominant role of men played in influencing the society as a whole. Opportunity for marriage is often mentioned as one of the main reasons for maintaining the practice of FGM [13,14]. Many women still belief that the chance of uncircumcised women to be married is very low and they are directly or indirectly forced to circumcise their daughters or support the practice. In a traditional society like Ethiopia or Somalia, marital decisions are mainly made by men or by the parent of the girl. In such society, men prefer marriage to circumcised women and mothers worry about their daughters thinking that an uncircumcised girl would not be married and becoming less attractive to men in terms of intactness. A study in Eastern Ethiopia depicted the preference of men to be married to circumcised woman [13]. This suggests that it would be very easy for women to abandon any type of FGM if the husbands do not expect it, indicating the importance of involving men in anti-FGM campaigns. Although men generally prefer to marry women who have undergone FGM, there are studies in some settings that have shown the preference of men to marry women who have not undergone the procedure [15,16].

In this study, men were found to be in favour of anti-FGM interventions (89%) compared with women (55%). Similarly, the intention to circumcise their daughter was lower among men (75.2%) than women (91.1%) respondents. This indicates that the practice of FGM is mainly supported by women and that men negative attitude towards the practice opens new possibilities to work against FGM. The higher response among men for the positive attitude towards anti-FGM interventions might be due to social desirability bias as the respondents wanted to satisfy the interviewers concerning the intervention, requiring a need to take care while generalizing the findings. However, men in our study were less knowledgeable about the consequences of FGM, highlighting the importance of taking into account when trying to involve men in anti-FGM interventions.

Our findings also indicate the difference between the types of FGM favoured by female circumcisers and what the respondents reported. About 63% of the respondents' revealed that partial or full clitoral cuttings were common, but 42% of the perpetrators reported such type of FGM, indicating the problem of under-reporting by the circumcisers about what they actually do. The Ethiopia DHS 2005 report indicates that about 74% of the women interviewed from Somali region were in favour of its continuation [5].

In contrast to our findings, study from Tanzania revealed that 76% of the circumcised women were in favour of not performing FGM on their daughters, while 24% did [14]. In Guinea, support for the continuation of FGM was significantly higher among women (68%) than among men (51%), indicating more attitudinal support for FGM discontinuation among men than women [17]. In a Sudanese village, 30% of young women and 53% of young men were intended to circumcise their daughter, whereas 50% of young women and 38% of young men were sure not to let their daughter undergo FGM [11].

Almost all of the practices of FGM in the present study had been performed by traditional circumcisers and birth attendants. According to the Ethiopia DHS 2000 report, more than 92% of the practices were performed by traditional circumcisers [4]. In Egypt, about 98% of FGM were performed by local circumcisers [8]. In Nigeria as well, most circumcisions were performed by herbalists and TBAs [18]. The majority of the traditional circumcisers in the present received trainings on anti-FGM interventions, but only few quitted the practice and the rest had continued it as a means of livelihood. This was a paradox in a sense that even if most of them were trained, they had continued practicing FGM and were using it as a means of income. A study from Guinea also identified financial gain and substantial gifts to the traditional practitioners of FGM in the community [12]. This might make the traditional circumcisers, who profit financially from the practice of FGM, resist anti-FGM interventions in their area, which requires efforts that should ensure and guarantee the traditional operators to get another means of income or opportunity through alternative skill training [1].

Our study identified and assessed the prevalence of FGM among girls ≤12 years of age. Studies involving such group of women, where such age range is optimal for FGM, are highly relevant to understand the current practices of FGM and help to design appropriate interventions. Thus age-specific rates for those being circumcised should be used when assessing the current prevalence of FGM. On the contrary, including a group of women in the age group of 15–49 years for assessing the prevalence of FGM in order to evaluate the impact of current interventions might not reveal the real situation of FGM in the community since those who had already been undergone the procedure could no longer be affected by anti-FGM intervention although their intention for subjecting their daughters to the practice and their attitude towards FGM could have been changed.

Although not well evidenced by the present findings, many villagers and circumcisers in the area indicated a shift of FGM practice from its severe form (infibulation) to the milder clitoral cutting, particularly since the last 5 years following the anti-FGM campaigns carried out in the area. However, cautious interpretation is needed because people may over-report about the shift by under-reporting the actual practice of FGM. A study conducted in Eastern Ethiopia has noticed the shift from infibulation to the clitoral cutting (*Sunni*) type among Adere and Oromo women after strong campaign by Muslim leaders favouring the milder type of *Sunni *circumcision [13]. Similar trend was also observed in other studies conducted in Sudan [11], Tanzania [14] and Nigeria [19]. Although the decline in the practice of infibulation is one of the indicators to assess the impact of anti-FGM interventions, advocacy for *Sunni *type of FGM shouldn't be encouraged while the world community is striving for eradication of any form of FGM.

Our study findings indicate some socio-economic determinants of the practice of FGM. The involvement of girl's parent in anti-FGM interventions was inversely associated with the practice or intention of FGM, indicating the need to intensify the interventions. However, cautious interpretation is needed because people with negative attitudes towards FGM might have a higher tendency to participate in the anti-FGM interventions. Although we found no association between education of the parents and the practice of FGM among girls, other studies showed that the level of education of women has a decisive role on the practice of FGM [9,11,19,20]. Illiteracy is one of the most important determining factors for the continuation of FGM. In Egypt, 92% of the mothers of circumcised daughters were illiterate compared with 69% of mothers of uncircumcised girls [8]. This is a good justification for considering education of women while implementing anti-FGM projects aiming to eradicate FGM.

Although this study provided much useful information, there were a number of limitations that can be subsequently addressed by other studies. First, the results cannot be generalized to other parts of Ethiopia where FGM is practiced, as the current study population are refugees originated from Somalia. The results are related to the Somali ethnic groups living in the Eastern Ethiopia and Somalia. Our study is also based on self-reporting of the parents. Although most of the studies on FGM-related practice are based on individual self-reports, through which respondents verbally describe their own or their daughter's status [8,11,12,21], the validity of the findings may be compromised. Because of the sensitive nature of FGM, it is difficult to know whether or not a girl is actually circumcised, and if so, with what type of FGM. A recent study from Sudan demonstrated poor reliability of reported form of FGM [22]. There was considerable over reporting of Sunni and under reporting of infibulation, the most severe form of FGM. Consequently, it is difficult to validate the women's response about the forms of FGM given without actually observing their genitalia, which obviously poses huge ethical and logistical concerns.

However, many studies have found no significant difference between self-reported and observed status of FGM, suggesting that assessing FGM through self-report may be a valid way of measuring the prevalence of FGM. In Nigeria, 45% of women were circumcised based on medical examinations, compared with 43.5% from self-reports [23]. There was a 97% agreement between reported circumcision status and that found on examination in the community-based study from Gambia [24] and 79% in urban and peri-urban Nigeria [19]. In rural Tanzania, consistency between self-reported and clinically detected FGM status was observed in about 80% of the women [20].

Whether FGM status based on self-reporting is valid depends on the context in which the questions are asked. If FGM is widespread, socially acceptable and considered as legal, then self-reporting is likely to be highly valid. If there are situations in which the respondents decline to reveal the truth that they are cut due to the fear of the national anti-FGM campaign under way or other reasons, other methods such as inspection of genitalia should be used to confirm the self-reports [9,14,18-20]. However, this will not be a major concern where high rates of FGM are reported, reflecting the actual status of the practice. In our study, under-reporting would not be a major problem since the reported prevalence of FGM was above 95% for girls in the age group of 11–12 years included in the survey. However, the validity of the information on the types of FGM might be difficult to differentiate based on the self-reporting of the mothers.

## Conclusion

The results of the present study revealed that the practice of FGM is widespread among the Somali refugee community, and there was a considerable support for the continuation of the practice. As revealed by the current study, participation in anti-FGM interventions is negatively associated with the practice and intention to circumcise the daughters. Thus, the present findings might stimulate further explorations on the prevalence and determinants of FGM on a wider scale. Finally, we recommend a continued dialogue with religious leaders and community members of the refugees to gain support in order to discourage and ultimately ban the currently widespread practice of FGM. The approach requires knowledge of Koran and the Islamic religion. In addition, community-based interventions about the possible consequences and the need to prevent FGM should be intensified particularly through involving men to bring about the desired change in behaviour of the local community.

## Competing interests

The authors declare that they have no competing interests.

## Authors' contributions

GM was the principal investigator of the study and took the leading role from conception and design to the final analysis and preparation of the manuscript. WD participated in the conception and design of the study, and took the leading role in data collection, analysis and final write-up of the study findings. All authors read and approved the final manuscript.

## Pre-publication history

The pre-publication history for this paper can be accessed here:


